# *RET*融合基因在非小细胞肺癌诊治中的意义

**DOI:** 10.3779/j.issn.1009-3419.2013.11.11

**Published:** 2013-11-20

**Affiliations:** 233004 蚌埠，蚌埠医学院第一附属医院肿瘤内科 Department of Medical Oncology, the First Affiliated Hospital of Bengbu Medical University, Bengbu 233004, China

**Keywords:** *RET*融合基因, 分子靶向治疗, 肺肿瘤, *RET* fusion gene, Molecular targeted therapy, Lung neoplasms

## Abstract

肺癌是当今世界癌症死亡的首位原因，分子靶向治疗是目前肺癌研究的热点。存在于部分肺癌亚群中的*RET*融合基因具有可识别的临床病理学特征，且RET抑制剂对其治疗有效，提示*RET*融合基因有可能成为该亚群患者个体化诊治的新靶点。本文将对*RET*融合基因的结构特征及其在非小细胞肺癌（non-small cell lung cancer, NSCLC）临床样品中的表达和治疗方面的研究进展进行阐述。

## Abstract (en)

Lung cancer is the leading cause of cancer-related death worldwide, molecular target therapy has become a hot research direction of non-small cell lung cancer (NSCLC) treatment. *RET* fusion gene with an identifiable clinical pathological features, is present in some subsets of lung cancer, and its treatment is effective by RET inhibitor, suggesting that *RET* fusion gene may be a new target for individualized treatment to the subgroup of NSCLC. This article reviews the structural characteristics of *RET* fusion gene and expression model in clinical samples, and treatment of NSCLC.

目前，肺癌的发病率和死亡率在全球范围内位居各类恶性肿瘤之首^[1]^，预计到2030年肺癌的归因死亡将占全球所有死亡原因的3.1%，这是一个惊人的数字^[2]^。非小细胞肺癌（non-small cell lung cancer, NSCLC）占肺癌的80%-85%，主要包括腺癌、鳞癌、大细胞癌、腺鳞癌、肉瘤样癌等，其中腺癌较易发生于女性及不吸烟者，近30年来所占比例有明显上升趋势。早期发现肺癌患者并给予及时适当的治疗，可以获得好的治疗效果^[3]^，然而80%以上的患者就诊时已失去手术根治的最佳时机，目前第三代含铂两药方案联合化疗是晚期NSCLC的标准一线治疗方案，但其疗效似乎已达平台：有效率仅为25%-35%、中位生存期为8个月-10个月、1年生存率为30%-40%^[4]^。

近年来随着肿瘤分子生物学的发展，人们逐渐认识到肺癌的发生、发展和转移是一个多阶段、多步骤、多基因参与调控的复杂的生物学过程。目前，通过寻找在肺癌发生发展过程中起重要作用的靶分子及信号途径，进行肺癌的分子分型及靶向治疗成为肺癌研究领域的热点。分子靶向治疗以其符合生理、低毒和理论上高效的特点，让人们看到了跨越这一平台的希望和曙光，是目前治疗晚期NSCLC最具前景的研究领域。

自2011年底开始陆续有报道RET（rearranged during transfection）融合基因存在于肺癌患者中，并且该基因具有可识别的临床病理特征，RET抑制剂抗肿瘤有效，提示*RET*融合基因可能是继表皮生长因子受体（epidermal growth factor receptor, *EGFR*）突变、*KRAS*（kirsten-rous avian sarcoma）突变、棘皮动物微管相关蛋白样4-间变淋巴瘤激酶（echinoderm microtubule associated protein like 4-anaplastic lymphoma kinase, *EML4-ALK*）融合基因后又一特异性较高的分子标记物。现将相关研究做一介绍。

## 1 *RET*融合基因

### 1.1 *RET*原癌基因与疾病

1985年Takahashi与Cooper等^[5]^在重组DNA的实验中，首次发现*RET*原癌基因。*RET*原癌基因定位于10号常染色体长臂（10q11.2），现已确定其DNA全长约8万个核苷酸（60 kb），有21个外显子，至少有4个转录产物，且在不同的组织中含量不同^[6, 7]^。由*RET*原癌基因编码的RET蛋白是一种受体酪氨酸激酶，为1, 114个残基跨膜蛋白，它有着受体酪氨酸激酶的经典结构：一个富含半胱氨酸的钙粘连素样细胞外区，一个跨膜区和一个催化酪氨酸激酶（tyrosine kinase, TK）的细胞内区。受体与配体结合后胞内区的TK磷酸化，激活下游信号转导通路，调节细胞生存并诱导细胞增生^[6]^。*RET*基因突变在多方面增强了RET TK信号转导的功能，进一步促使激酶的活化和原癌基因的转化^[8]^。*RET*原癌基因能与多种基因发生融合，常以本身断裂再与另一基因接合的方式重组成一个新的基因（融合基因），从而逃脱受体结合配体的调控，进行自我磷酸化、自动传导信号，引发肿瘤生成。

之前的研究提到*RET*基因突变是甲状腺癌症的诱因，不同形式的染色体异位和插入导致*PTC*/*RET*融合基因的形成，被认为是甲状腺乳头状癌（papillary thyroid carcinoma, PTC）的驱动突变^[9]^。随着研究的不断深入，已经发现*RET*基因突变与多种疾病的发生密切相关，包括多发性内分泌腺瘤2型^[7]^、PTC、甲状腺髓样癌（medullary thyroid carcinoma, MTC）^[10]^、先天性巨结肠^[11]^和近期发现的肺腺癌（lung adenocarcinomas, LADCs）^[12]^等。

### 1.2 *KIF5B*（kinesin family member5B）基因

*KIF5B*基因位于10号常染色体短臂（10p11.22），编码KIF5B蛋白，该蛋白是Kinesin驱动蛋白家族1的成员，含有约350个氨基酸残基，是由单体组成的同四聚体，内有三磷酸腺苷（adenosine triphosphate, ATP）结合位点和微管结合位点，其“头部”具有ATP酶活性，能通过水解ATP获得能量，改变构型，且只能以微管构成的轨道朝一个方向滑行。KIF5B的蛋白结构域—LC-S6在启动基因融合事件中不可或缺，它具有一个蜷曲螺旋结构，可以沿着细胞微管运动，使RET蛋白聚合从而导致自体活化，自发诱导下游磷酸化信号转倒。KIF5B和RET通过臂间倒位后融合形成*KIF5B-RET*融合基因^[13]^，导致多种肿瘤生成。

### 1.3 *CCDC6*（coiled-coil domain-containing protein 6）基因

*CCDC6*基因位于10号常染色体长臂（10q21），编码的卷曲螺旋结构域蛋白6参与形成细胞骨架结构。*CCDC6-RET*基因常见于甲状腺癌样本中，它的过表达会导致肿瘤的发生，被认为是*PTC/RET*的典型驱动突变基因^[9]^。也有报道^[14, 15]^指出*CCDC6*和*RET*的融合可能导致LADCs的发病。

### 1.4 *TRIM33*（tripartite motif-containing 33）基因

也称RFG7或TIF1，位于1号常染色体短臂（1p13.1），是转录中介因子家族的成员之一，控制参与细胞分化^[16]^。已有报道^[17]^证实TRIM33和RET的融合与PTC的致病有关，我们期待能够证实*TRIM33-RET*基因在NSCLC中也有临床意义。

### 1.5 *NCOA4*（nuclear receptor coactivator 4）基因

*NCOA4*基因位于10号常染色体长臂（10q11.2），在睾丸、肾上腺、甲状腺、胸腺、前列腺等多种组织中广泛表达。近期有报道^[18]^在NSCLC中发现1例NCOA4-RET阳性患者，提示*NCOA4-RET*融合基因或许也对NSCLC有临床意义。我们相信随着研究的不断深入，未来会发现更多有意义的新的融合位点。

## 2 *RET*融合基因的检测方法

目前已报道的常用*RET*融合基因检测方法包括免疫组化（immunohistochemistry, IHC）、荧光原位杂交技术（fluorescence in situ hybridization, FISH）、聚合酶链式反应（polymerase chain reaction, PCR）等，其中PCR又包括逆转录-聚合酶链式反应（reverse transcription-polymerase chain reaction, RT-PCR）、cDNA末端快速扩增技术（rapid amplification of cDNA ends, RACE）和DNA测序技术。多数学者采用RT-PCR法进行检测，也有采用FISH检测，但目前仍无敏感特异稳定的方法用于该分子的检测。研究证明FISH与RT-PCR的检测结果一致，但与IHC的匹配欠佳^[19]^。这提示前二者可能是检测*RET*基因状态的最佳选择，且各有优势。我国陈海泉教授及其团队通过不断摸索，创造发明了一种高效、准确、低廉的检测方法——“基于表达不平衡的*RET*融合基因检测”。目前，这种检测方法及其主要技术要素已申请国家发明专利，有望得到进一步推广和应用。

## 3 *RET*融合基因在NSCLC中的表达及其临床病理学特点

我国研究者对中国LADCs人群进行基因突变筛查的结果显示：*EGFR*突变率为66.3%，*KRAS*突变为2.3%^[20]^。国外研究^[21]^提示LADCs中*EGFR*基因突变率为15%，*KRAS*基因突变率约为25%，*EML4-ALK*融合基因突变率约为5%，其他约15%，至今仍有约40%的基因突变尚不明确。2011年12月，韩国Ju等^[22]^在1例肺癌患者身上首次发现*KIF5B-RET*融合基因，随后陆续出现对*RET*融合基因进行研究和报道，提示肺癌中存在新的分子亚型—*RET*融合基因型肺癌。*RET*基因与其他基因融合已被证实是甲状腺癌症的诱因^[9, 10, 17]^，现有研究提示它在肺中表达水平低，但是在患者肺中高表达，*RET*融合基因可能成为LADCs的新驱动基因。

韩国Ju^[22]^等应用转录组高通量测序技术对1例33岁、无癌症家族史、从不吸烟的男性肺腺癌肝转移灶的标本进行检测，发现了一种新的融合基因：*KIF5B-RET*，为KIF5B的16号外显子与RET的12号外显子相融合，其肿瘤组织EGFR、KRAS、EML4-ALK均为阴性。随后对20例LADCs样本进行定向测序分析，再次发现2例*KIF5B-RET*融合基因阳性患者（5例EGFR、KRAS、EML4-ALK均为阴性的LADCs患者中发现1例；15例EGFR、EML4-ALK均阴性的患者中发现1例），阳性率约为6%（4.3%-8%）。*RET*基因的12号外显子分别与KIF5B的15、23号外显子相融合，这种融合可导致*RET*原癌基因的高表达。这证实了KIF5B-RET融合型NSCLC的存在，并提示该融合基因可成为肺癌个体化诊治中新的分子靶点。

日本Takeuchi等^[23]^经分子和组织病理学检测1, 529例肺癌（其中1, 116例为LADCs）的融合基因表达情况，采用IHC或FISH技术分别分析了*ALK*、*ROS1*（sarcoma virus oncogene homolog 1）、*RET*这3种融合基因的频率分布。共检出71例肺癌患者存在融合基因，其中44例*ALK*、13例*ROS1*和14例*RET*（12例为*KIF5B-RET*融合，2例为*CCDC6-RET*融合）。这71例病理类型均为LADCs，即0.9%肺癌（1.2% LADCs）患者存在*RET*融合基因，且研究发现*RET*基因的断裂位点相对保守，14例阳性标本中11例位于12外显子，1例位于11外显子，1例未知。该研究中*RET*融合基因阳性患者EGFR、KRAS为阴性，具有年轻、少吸烟、肿块小的特点，其总生存期（overall survival, OS）与*EGFR*突变阳性患者相比无明显差别（*P*=0.32）。该研究通过这71例融合基因确定了4个独立的不良预后因素：年龄≥50岁、男性、高病理分期、融合基因阴性。

日本Kohno等^[12]^对319例日本肺腺癌标本行RT-PCR和Sanger测序分析显示，6例（1.9%）KIF5B-RET阳性均为非吸烟腺癌，癌组织高表达RET，肿瘤病理类型均为中分化或高分化，同时EGFR、KRAS、人类表皮生长因子受体2（human epidermal growth factor receptor-2, HER-2）及ALK均为阴性。*RET*融合基因存在4种变体（K15：R12，K16：R12，K23：R12，K24：R8）。但是，研究者对80例美国肺癌患者及34例挪威肺腺癌患者进行检测时，仅从1例美国曾经吸烟肺腺癌患者中发现了*KIF5B-RET*基因融合。*KIF5B-RET*融合基因在亚裔和非亚裔LADCs中阳性率为1%-2%，而在其他肺癌类型包括234例鳞状细胞癌、l7例大细胞癌、20例小细胞癌中均未检测到*KIF5B-RET*融合基因；在其他肿瘤的腺癌中，例如卵巢癌（*n*=100）、结肠癌（*n*=200）亦未检测到*KIF5B-RET*融合基因，这一结果提示*KIF5B-RET*融合基因为LADCs所特有。且*RET*基因的断裂位点相对保守，以上7例RET阳性样本中6例位于12号外显子，1例位于8号外显子。

美国Dana Farber癌症研究所Capelletti等^[24, 25]^通过二代测序技术检测24例肺癌的常规石蜡包埋组织标本，发现了1例KIF5B-RET融合变异。另外利用IHC技术在117例NSCLC患者中检测到22例RET阳性，对其中15例患者进行测序发现1例携带*KIF5B-RET*融合基因。检测另外526例（121例白人和405例亚裔）从不或很少吸烟的肺癌患者的融合基因情况，共发现1例白人（0.8%）和9例亚裔（2%）为KIF5B-RET阳性，且所有10例*RET*融合基因阳性患者均无EGFR、KRAS、Her-2、ALK、ROS1的改变。Capelletti研究的667例肺癌标本中*KIF5B-RET*基因融合频率约为1.8%（12/667），在没有EGFR、KRAS、Her-2、ALK、ROS1变异的肺癌中，*RET*基因的融合频率高达6.3%（10/159例）。该研究中发现4种*KIF5B-RET*融合基因的变体（K15：R12、K16：R12、K22：R12和K15：R11），可以看出*RET*基因的断裂位点较为保守。

Suehara等^[26]^综合应用NanoString、RACE和RT-PCR在74例KRAS、EGFR、HER-2、BRAF（murine sarcoma viral oncogene homolog B1）均无突变且*ALK*融合基因为阴性的LADCs中检测出2例KIF5B-RET阳性患者，其中1例为60岁从不吸烟的女性患者，1例为73岁曾经吸烟的男性患者。

日本Yokota等^[27]^采用RT-PCR技术及直接测序法对442例术后患者（270例肺腺癌、101例肺鳞癌、60例乳腺癌、11例来自结肠癌和PTC的转移性肺癌）的*KIF5B-RET*基因状态进行研究，结果检出3例KIF5B-RET阳性且均为LADCs（1.1%），其他172例肺鳞癌、乳腺癌、转移性肺癌的*KIF5B-RET*融合基因均为阴性。这3例KIF5B-RET阳性患者均为女性，无吸烟史、无*EGFR*、*KRAS*、*BRAF*、*ERBB2*、*EML4-ALK*基因变异。

我国季红斌课题组^[14]^在前期工作中已经揭示了绝大多数LADCs中存在关键致癌驱动基因。只有少数LADCs（24/202）的关键致病基因仍在进一步探究：选取5例EGFR、HER2、KRAS、EML4-ALK均为阴性和12例致癌驱动机制已明确的肿瘤样本进行Affymetrix外显子芯片的分析及实验验证，最终在这些非吸烟LADCs标本中检出1例*CCDC6-RET*融合基因，并且进一步证实为CCDC6的1号内含子和RET的11号外显子在染色体DNA水平的转位所致。RET是一个受体酪氨酸激酶，在正常的肺组织中表达较低；而融合了CCDC6胞外域以及RET激酶活性区的突变基因，在肺癌样本中往往表达较高，可能是导致肺癌发病的关键所在。

我国复旦大学癌症中心的王瑞博士与陈海泉博士等^[18]^应用PT-PCR结合实时定量PCR的方法对936例术后NSCLC患者的*RET*融合基因情况进行研究，再通过IHC和FISH对所得结果进行验证。检测到13例患者（633例腺癌患者中有11例，24例腺鳞状细胞癌患者中有2例）存在*RET*融合基因，其中9例为*KIF5B-RET*，3例为*CCDC6-RET*，1例为首次发现的*NCOA4-RET*融合基因。*RET*融合基因阳性的LADCs患者，平均无复发存活期为20.9个月，其肿瘤分化情况较差（63.6%, RET *vs* ALK, *P*=0.029; RET *vs* EGFR, *P*=0.007），患者年龄偏低（≤60岁；72.7%），不吸烟者（81.8%）多发，并且多见实体亚型（63.6%）、瘤体较小（≤3 cm）、有N2病情（54.4%）。

Drilon ^[28]^报道了在临床研究中应用新一代测序法首次发现1例*TRIM33-RET*融合基因阳性的NSCLC患者，并进一步证实为TRIM33的14号外显子和RET的12号外显子相融合，且无*MET*基因突变。

目前已报道的研究显示我国*RET*融合基因存在于1.4%的NSCLC患者以及1.7%的LADCs患者中^[18]^，国外数据为*RET*融合基因占NSCLC患者的1%-2%^[28]^（其中KIF5B-RET占90%，相继发现的CCDC6-RET、NCOA4-RET、TRIM33-RET数量较少，尚不能得出有力结论），并且该基因具有可识别的临床病理特征，与其他已知的NSCLC分子亚型相互独立、不相重叠，因此可定义为一项全新的分子亚型，对其进行靶向治疗研究，进一步应用于临床工作。

## 4 RET抑制剂的抗肿瘤作用

已有研究^[29]^表明*RET*融合基因可诱导甲状腺癌的发生，给予RET抑制剂后肿瘤可得到控制，然而在肺癌中相关报道较少。

多靶点激酶抑制剂凡德他尼（vandetanib）是血管内皮生长因子受体（vascular endothelial growth factor receptor 2, VEGFR-2）、EGFR和RET信号通路的抑制剂。一项凡德他尼治疗局部晚期或转移性家族遗传性MTC的Ⅱ期临床试验^[29]^结果表明，疾病控制率达到73%，且不良反应可以得到控制；另一项关于MTC的Ⅲ期临床试验（ZETA）^[30]^结果显示，凡德他尼组较安慰剂组无进展生存期（progression-free survival, PFS）明显延长，疾病进展风险降低54%。因此，凡德他尼已被美国食品药品管理局批准用于不宜手术切除或转移性MTC的治疗。众多学者对于凡德他尼治疗RET融合基因阳性的NSCLC患者是否有效进行了研究。

Kohno等^[12]^对仅表达*KIF5B-RET*融合基因而无其他基因变异的H1299人肺癌细胞进行研究，检测到KIF5B-RET蛋白在RET激酶活化环上Tyr905发生磷酸化，证实了KIF5B-RET的融合导致RET激酶异常活化。研究发现凡德他尼（< 1 μmol/L）能够抑制转染表达*KIF5B-RET*融合基因的NIH3T3细胞模型生长及体外克隆形成，提示该药能够抑制Tyr905的磷酸化，抑制*KIF5B-RET*融合基因的活化。Takeuchi等^[23]^将转染了KIF5B-RET病毒的3T3细胞接种于裸鼠皮下，可观察到肿瘤形成。凡德他尼能够抑制转染了KIF5B-RET病毒的Ba/F3细胞增殖。Capelletti等^[24]^进一步发现，转染了KIF5B-RET变体K15：R12的Ba/F3细胞显示出RET的高表达和磷酸化活化，体外实验发现，多靶点激酶抑制剂舒尼替尼（sunitinib）、索拉非尼（sorafenib）和凡德他尼均能够抑制KIF5B-RET Ba/F3细胞的增殖，且舒尼替尼能够抑制RET磷酸化，但EGFR-TKI吉非替尼（gefitinib）无以上作用。

目前两项凡德他尼单药治疗NSCLC的Ⅲ期临床试验正在进行中。关于凡德他尼与厄洛替尼（erlotinib）的对照试验—ZEST试验^[31]^结果显示两组PFS、客观有效率（objective response rate, ORR）和OS均无明显差异。Lee等^[32]^设计的ZEPHYR试验将符合条件的患者按2:1的比例随机分到凡德他尼组（300 mg/d）和安慰剂组，接受治疗直至疾病进展或发生不能耐受的毒性反应，该研究的主要目标为OS，最终纳入924例患者，凡德他尼组617例，安慰剂组307例。结果显示，常见的不良事件凡德他尼组多于安慰剂组，包括腹泻（46% *vs* 11%）、皮疹（42% *vs* 11%）和高血压（26% *vs* 3%）。8周时凡德他尼组PFS（危险比=0.63，*P* < 0.001）和ORR（2.6% *vs* 0.7%, *P*=0.028）均优于安慰剂组；然而两组的中位OS无差异（8.5个月*vs* 7.8个月，*P*=0.527），凡德他尼组PFS的延长未能最终转化为OS的获益。因此，我们期待一项新的随机、双盲、多中心临床试验出现，将筛选出的*RET*融合基因阳性患者，随机分到凡德他尼组和对照组进行研究，在试验的初期更精确的锁定治疗靶点，或许能够得出更有意义的结果。

Cabozantinib即XL184，是一种口服广谱激酶抑制剂，通过靶向抑制MET（mesenchymal-epithelial transition factor）、VEGFR2及RET信号通路而发挥抗肿瘤作用，它能够杀死肿瘤细胞，减少转移并抑制血管生成^[33]^。研究^[33]^表明Cabozantinib对于RET-PTC的细胞株（IC_50_为0.06 μM）治疗效果优于阿西替尼（Axitinib）、舒尼替尼、凡德他尼，鉴于此斯隆凯瑟琳癌症中心启动首个Cabozantinib治疗KIF5B-RET阳性的进展期NSCLC患者的Ⅱ期临床试验（NCT01639508）。Drilon等^[28]^对此项研究进行了报道，该试验共招募25例符合条件的患者接受口服Cabozantinib 60 mg/d治疗，连续28天为一个周期，直至疾病进展或发生不可耐受的药物相关毒性。该研究主要目标为ORR，次要目标为PFS、OS和不良反应，目前第一阶段已顺利完成，3例经过Cabozantinib治疗的患者中有2例达到部分缓解（RECIST 1.1评价标准）：其中1例为*TRIM33-RET*融合基因阳性患者（这也是首例被报道的TRIM33-RET阳性的NSCLC患者）；另外1例KIF5B-RET阳性患者达到稳定。这3例患者目前仍在继续接受治疗，此项研究预计最终于2015年7月全部完成。

## 5 结语及展望

分子分型的出现为晚期NSCLC的治疗开创了全新局面。*RET*融合基因成为NSCLC的一个新的分子亚型，有着独特的临床病理学特征：多为不吸烟（或少吸烟）的较年轻的腺癌患者，其肿瘤分化情况较差，瘤体较小，有N2病情，与其他已知的基因改变不共存，RET抑制剂治疗有效。虽然*RET*融合基因在肺癌中的发生率很低，但由于肺癌在全世界高发，如能结合临床病理学特点，对*EGFR*突变、*KRAS*突变、*EML4-ALK*基因均阴性的肺腺癌患者进一步行*RET*融合基因检测，并对阳性患者进行针对性靶向治疗，相信会使更多患者获益。

在肺癌中，*RET*融合基因的药物临床试验及分子诊断技术方法成为新的研究热点，RET融合型肺癌的诊治模式已经初露端倪。当然，真正形成类似目前*EGFR*突变型、*ALK*融合型肺癌的诊治模式，还需要高水平、大规模、前瞻性临床试验的证据支持。目前所检测到RET融合基因阳性的肺癌几乎均为腺癌，类型较为单一，但由于相关研究较少，其他类型肺癌中是否存在RET融合基因、是否还存在其他基因与*RET*基因融合的现象都有待进一步的探索。多靶点激酶抑制剂凡德他尼、克唑替尼、Cabozantinib等能否成为该分子亚群肺癌治疗的里程碑，也有待更多更深入的实验室研究、转化性研究及大规模前瞻性多中心随机对照临床研究加以佐证。但是我们相信不久的将来会有相应的药物给肺癌患者带来新的希望。
